# Playing online videogames—more than just entertainment? A qualitative study of virtual social participation in persons with spinal cord injury

**DOI:** 10.3389/fresc.2024.1395678

**Published:** 2024-05-16

**Authors:** R. Nilsen, T. Johansen, M. Løvstad, A. M. Linnestad

**Affiliations:** ^1^ Department of Research, Sunnaas Rehabilitation Hospital, Nesodden, Norway; ^2^ Department of Rehabilitation Science and Health Technology, Faculty of Health Science Oslo, Oslo Metropolitan University, Oslo, Norway; ^3^ Department of Psychology, Faculty of Social Sciences, University of Oslo, Oslo, Norway; ^4^ Department of Research, Lovisenberg Diaconal Hospital, Oslo, Norway

**Keywords:** qualitative, SCI, spinal cord injury, social participation, virtual social participation, video games, gaming

## Abstract

**Introduction:**

Spinal cord injury (SCI) affects many aspects of life, physically, emotionally and socially. Engaging in online videogames holds the potential to facilitate increased social interactions for individuals with SCI. The aim of this study is to increase our understanding of the experiences people with SCI have with using online videogames as an arena for social participation.

**Methods:**

A focus group interview was conducted with seven participants with SCI, aged 15–35, all experienced in using online videogames as a method of socializing. The data was analyzed using thematic analysis.

**Results:**

The participants highlighted that playing online videogames way of maintaining social connections and expanding their social network. However, they faced challenges due to limited knowledge and negative attitudes from others regarding use of videogames as a social arena. Three main themes were developed from the findings: “Disabling social barriers”, “Attitudes towards gaming” and “Gaming—connecting people”.

**Conclusion:**

Healthcare professionals should consider videogames as a leisure activity and facilitate their use, recognizing their potential for social interaction and well-being. Prioritizing activities that promote social interaction is crucial for good health.

## 1 Introduction

A spinal cord injury (SCI) may affect many aspects of life, physically, emotionally and socially. Functional disability after SCI thus goes beyond medical characteristics and may also affect social and interpersonal relations (1). Factors such as physical environmental barriers, pressure ulcers, challenging bowel and bladder function and urinary tract infections can lead to restricted participation in everyday activities (2). These barriers can result in involuntary loneliness (3). Loneliness is associated with an individual's experience of their own social interactions, which is an essential component of good health and well-being (4). A Norwegian public health report from 2018–2019 emphasized that prevention of loneliness can contribute to improved quality of life and promote good mental health (5).

Social participation is a multi-dimensional construct, and there is no universally agreed upon definition (6). However, Levasseur and colleagues define social participation as a person's involvement in activities that provide interactions with others in the community and in important shared spaces. Furthermore, social participation is shaped by available time and resources as well as societal context and individual preferences (7). This definition is based on literature on aging adults, but can apply to other populations as well. In addition to their definition, Levasseur and colleagues have proposed a taxonomy in which participation is seen as being on a continuum of six levels (8), and where the last four, are considered to constitute social participation: interacting with others without physical contact, doing an activity with others, helping others and contributing to a community. This taxonomy highlights how various aspects of social participation are related to each other (8), and notably, all of these may be involved when playing videogames online.

Playing videogames is a popular leisure activity worldwide, and the number of people who report playing videogames has never been higher (9). So far, investigations on the therapeutic use of videogames within healthcare has focused on its effectiveness regarding physical functioning (10–12). However, playing online videogames has emerged as an important arena for social participation for many individuals (13).

Conventionally, playing videogames has typically been considered as an activity that can lead to aggression and social isolation, which might have negative effects on somatic health. Such negative perspectives still exist in scientific research (14–18). In addition, mass media often still portrays the typical gamer as a socially isolated or even violent person (19). Recent research has, however, provided a more positive approach, showing an association between playing videogames and better mental health and well-being and demonstrating that adolescent social gamers report less loneliness than people in the same age group that do not play videogames (9, 20, 21). De la Hera emphasizes that playing online videogames can be a way of reinforcing family bonds and reducing social anxiety (22).

The majority of newer videogames include social features enabling playing and communicating with others online (23). In addition, social media platforms such as Discord or Twitch can be used to facilitate communication while playing videogames online (23). Duchenaunt and More reported that videogames could be used as a method for social participation, and Kleban and Kaye stated that it can be seen as a tool to increase social networks (24, 25). Interacting with others through videogames thus has the potential to have a positive impact on mental health. Furthermore, the use of online videogames has been described as a valuable technical aid in enabling social participation in situations where physical gatherings are challenging (26). People living with disabilities have emphasized that within a virtual world, their physical disability is not visible (25).

Computer technology is constantly evolving, and the use of videogames is playing an increasingly important role in rehabilitation after injury or illness (27). It is therefore important to increase our knowledge about the potential for social use of videogames in the rehabilitation process. Guilcher and colleagues pinpointed that it should be a major objective for healthcare professionals in rehabilitation to facilitate social interaction and participation in society and thereby promote good health and well-being (2). A review of the literature revealed that studies examining the experience of the social aspects of playing videogames are limited, and studies investigating how persons with disabilities experience socializing through videogames are almost non-existent. We have not been able to identify any studies that have investigated how persons with SCI experience the use of videogames as a social arena. The aim of this study is to increase the understanding of the experiences people with SCI have with using online videogames as an arena for social participation.

## 2 Materials and methods

The social constructivist epistemological worldview was applied in this study, which allowed for an exploration of knowledge that is socially constructed. This perspective enabled gathering experiences from individuals to describe the complexity of virtual social participation (28). Social constructivism is based on the foundation that knowledge is created through human activity and that reality is created jointly by members of a society. Furthermore, learning is seen as an active and social process that occurs when individuals engage in social activities containing interactions and collaboration (29). The theoretical framework for this study was chosen in order to bring depth to important perspectives and to guide how the collected data was read, interpreted and discussed (30). Maslow's hierarchy of needs was applied to understand social interactions as a basic need for well-being (31). Social interaction fulfils fundamental need for connection and belonging. Socializing plays a significant role in nurturing our self-worth and fostering personal growth. In various contexts, through friendships, group activities or collaborative pursuits, social interaction aligns with Maslow´s fundamental human needs. Furthermore, as lack of social interactions may represent a risk of poor health, the salutogenic approach was applied to bring a deeper understanding to how virtual socialization can be a resource to promote good health (32).

### 2.1 Participant recruitment

The study utilized a combination of purposive and convenient sampling strategies to recruit participants (33). Inclusion criteria included having a verified SCI and self-reporting of using online video games as a method of social participation. No selection criteria was set regarding age, gender or socioeconomic status. Target sample size was ten participants, in line with existing recommendations (34). Participants were recruited either through an ad campaign in social media or by identification by healthcare professionals at a specialized Rehabilitation Hospital in the South East region of Norway. Contact with participants was initially established through a phone call, during which further information was provided. All participants provided written informed consent. Nine participants were originally recruited, one of which was a woman. Two participants withdrew close to the interview and the final sample consisted of seven male participants with different levels of SCI.

### 2.2 Study and environmental context

The present study was conducted during the Covid-19 pandemic, hence there were several social restrictions that affected the study procedures, data collection and participant recruitment. The data collection was performed in an online semi-structured focus group interview via the Norwegian health administration's conference call solution (join.nhn.no) since meeting in person was prohibited at the time of the interview.

### 2.3 Procedure

The focus group interview, facilitated by the author RN, lasted approximately 90 min as suggested by Krueger (34). In addition to the interview phase, the 90 min contained presentation of the interviewers and the participants involved, technical formalities regarding the data collection, and final remarks from the participants. Author TJ was responsible for taking notes during the interview, noticing participant comments through a show of hands and for contributing follow-up questions as needed. RN had experience in performing qualitative interviews and RN and TJ had preparation meetings with author AML before the focus group interview. Author AML also has experience in conducting qualitative interviews. In the meeting, possible challenges such as conflicts, privacy and dominant participants were addressed. The interview followed a semi-structured guide developed by RN and TJ with guidance by AML prior to the interview. Author AML did not partake in the interview setting. Open-ended questions were asked and the interviewer was cautious not to evoke biased responses. Follow-up questions were asked to gather different points of view from the participants. The interview guide consisted of two main sections. The first section focused on the participants' experience with gaming, both in general and in gaming with a disability. Including questions such as “when did you start playing videogames?”, “what experiences did you have with playing videogames prior to your injury?” and “what type of videogame equipment do you use?”. The second section focused on the participants' thoughts regarding online videogames as a method of socialization, and included questions such as “how have you used videogames as an arena for socialization?”, “with whom do you play?” and “how does socializing through videogames compare to face-to-face interactions?” The interview was shared video, however only audio was recorded. The recording was transcribed verbatim, which led to thirty pages of text.

### 2.4 Data analysis

The data was analyzed using reflexive thematic analysis following the six-step process of analysis described by Braun and Clark (35). The analytical process was performed by authors RN and TJ, taking an inductive approach (35). Familiarizing with the data was performed by reading the transcription multiple times, both individually and together. Then, the process of coding was initially performed by RN and TJ and further discussed with AML. After discussion the codes where agreed upon and initial themes were generated. The themes were reviewed multiple times and further developed in meetings with AML. The themes were refined, defined and named according to content and meaning. The draft for the report was written by RN and TJ, and reviewed and refined by AML and ML. To ensure validity and reliability of the data and analysis, strategies proposed by Creswell & Creswell were applied (36). Hence, author AML checked the coding multiple times for consistency, created separate transcriptions of the interviews and performed external auditing of the codes.

According to Braun and Clarke, the thematic analysis is characterized as reflexive, where the epistemological framework guides the reading and analysis of the data (35). In line with an inductive approach, the codes were created first and theoretical perspectives were subsequently applied in order to guide the following inductive analysis. Relevant studies were then identified to support and contextualize the experiences of the participants.

### 2.5 The researcher's role and preconceptions

The researcher's role is influenced by their preconceptions, and Malterud describes preconception to encompass experiences, claims, ideas, professional perspectives and epistemological grounding (37). The research aim of the study was based on two of the authors' (RN and TJ) interests as well as their professional background as occupational therapists at a specialized rehabilitation hospital. Three of the authors have an MPhil degree (RN, TJ and AML), while the fourth (ML) is a professor in psychology. TJ and AML are currently PhD students. Two authors (RN and TJ) have regularly used videogames and virtual reality as a rehabilitation tool for physical exercise, pain management and as a leisure activity. None of the authors had been primary therapists for any of the participants during their post-acute rehabilitation process. During this study, the authors were cautious not to allow their preconceptions affect the work to ensure reliability and validity of the research (37, 38).

## 3 Results

All seven participants were male. They ranged in age from teenager to their early thirties and lived in different parts of Norway. All participants used a wheelchair for mobilization. Some had lived with their SCI since childhood, while others had acquired their injury over the last few years. Due to the different levels of SCI, the participants had varying levels of physical functioning. Four participants had paraplegia, and three had tetraplegia. Hence, a few used adaptive controllers, while others used standard gaming equipment. All participants had played videogames since they were children and had various experiences with socialization through videogames since before their injuries. Some participants had primarily played with friends and siblings while others had competed in e-sports.

### 3.1 Findings

The analysis revealed three major themes: “Disabling social barriers”, “Attitudes towards gaming” and “Gaming—connecting people”.

#### 3.1.1 Disabling social barriers

The theme “Disabling social barriers” encompasses the experiences of participants who expressed a desire to be social, but were prevented from being so due to both physical and mental barriers. In addition, the theme includes how playing videogames has been an alternative form of socialization for these participants.

The participants described that they used videogames as an arena for socialization during times when meeting face-to-face was difficult due to various barriers. The participants commonly recognized that playing online videogames could be used as an alternative to physical social participation. They shared experiences about dealing with physical barriers such as neurological pain, lack of wheelchair accessible environments and long hospital admissions. One participant stated that instead of receiving flowers while hospitalized after incurring a SCI, his friends bought him a laptop. Because of this, he was able to communicate and virtually interact with family and friends through playing online videogames. The participant expressed a need to be social and to keep in touch with his social network at home, especially because he was in a situation where his life had radically changed. Many of the participants experienced the same need, but during hospital admissions, healthcare professionals put little focus on encouraging playing videogames as an arena for keeping in touch with family and friends. Additionally, the participants stated that they were unable to play videogames at the hospital. Some of the participants stated that they had to bring their own gaming equipment to the hospital to be able to play videogames. Even then, there were issues regarding internet connectivity. Hence, playing online videogames in this environment was a challenge.

The participants also talked about how a lack of inclusion could be a barrier for social interaction. One participant described facing barriers when he had a desire to participate in social activities, but was unable to do so due to other people's negative attitudes towards disabilities.

When I was younger, some of my friends had parents that didn't want me playing at their house because I was in a wheelchair, and they didn't want to be responsible for me. (…) So I spent most of my time playing videogames with people from the US (Participant 1).

The quote suggests that others' negative attitudes regarding disabilities and the need for technical aids could hinder social participation and that videogames were used as an alternative method of socialization. Several other participants had experienced similar incidents, and they agreed that such attitudes and actions were quite common.

The participants also described that the feeling of imposing extra work or responsibility on friends and family when participating in physical activities could be a reason why they choose not to socialize as frequently. They explained that participating in various activities where they would need help and accommodation could lead to feeling like a burden:

If your friends decide to go swimming, you might want to join. But then you feel that you are somewhat a burden because you may need some help. But, if we meet online, everyone is at the same level. You don't have to think that you're the weak link—we are all equal (Participant 7).

The quote indicates that it can be uplifting to engage in an activity where everyone can participate on equal terms despite having different functional abilities. The participant felt that he did not have a handicap in the virtual world. Furthermore, the participants noted that they did not have the same abilities as their friends and family when it came to participating in some physical activities. However, they expressed pride and self-confidence in being able to excel in other areas: “*I am easily beaten in the 60-meter sprint at school. But in a videogame that I am good at, people don’t have a chance”* (Participant 2).

#### 3.1.2 Attitudes towards gaming

The theme “Attitudes towards gaming” presents the participants' encounters with negative and positive attitudes related to the use of videogames as a social activity, as well as experiences the participantś themselves had with videogames as an arena for socialization.

The participants shared that over the years, they had encountered negative attitudes related to the use of videogames. They believed that a lack of understanding of how videogames can be a social activity might be the reason that negative attitudes still exist.

My mother has throughout my childhood always said that playing videogames only makes me less social. They (parents) are more used to face-to-face interactions because that was all they had. So of course, when they look at a person that's playing a videogame, they're going to assume that he is antisocial. They don't think about the fact that sometimes online interaction is all you have (Participant 1).

The quote indicates that socialization through videogames may not be valued in the same way as face-to-face socialization. The participant expressed that sometimes he was not able to meet others physically and therefore chose to socialize via videogames. Those around him did not always understand that in playing videogames with others, he was in fact socializing, even though they were not physically sitting in the same room.

Several participants further shared that they experienced different demands and expectations for virtual socialization as opposed to face-to-face socialization. They explained that many did not understand that virtual socialization does indeed set expectations of attendance, performance and communication just like physical encounters:

My parents have asked me if I could pause an online game. They do not understand what it's about. You're playing with people from all over the world, competing, and it actually means something to win. (…) You don't just ruin the game for yourself if you leave. Yes, it's online, but it's with people and it’s not just sitting alone with a screen. You talk and communicate with people. It's like bowling with your friends—you're doing an activity with others (Participant 5).

Other participants had experienced similar misconceptions and compared this experience to pausing a football match in the middle of the game. The quote suggests that other people did not fully understand what online videogames entails. They fail to realize that it includes several partakers, and the player is part of a community that allows for socialization on a virtual platform.

Despite the fact that the participants had experienced a lack of understanding from others, many of them had noticed a change in attitudes related to videogames and virtual socialization during the last few years. The participants felt that the Covid-19 pandemic had played a positive role in this change of attitude. Because of the pandemic, more people experienced a lack of face-to-face socialization due to restrictions in society: “*Covid-19 has done a lot for (understanding) videogames, and people have opened their eyes to gaming as a way of socializing because they have tried it themselves”* (Participant 6).

#### 3.1.3 Gaming—connecting people

The theme “Gaming—connecting people” encompasses the participants' experiences with videogames as a way to maintain contact with friends and family, make new connections and expand their social network.

Participants gave several examples of how videogames could be a positive factor in staying in touch with friends and family. The participants stated that through playing videogames, they were able to reconnect with people they had not spoken to in a long time.

Some participants used videogames to maintain contact with existing friends and family, old classmates or work colleagues. In addition, several participants used online videogames as a way to get to know new people and stated that they had expanded their social network. One participant described how online communication over several years of playing videogames with others could result in a feeling of mutual friendship:

I have pretty good friends that I have known for almost 10 years, but that I have never met (face-to-face). People from America, Canada, Portugal and Germany. (…) We have played together for so many years—it feels like we have developed a real friendship. So for me, gaming is definitely social (Participant 4).

The quote shows that the participants also made connections outside Norway, despite not having met face-to-face. The participant further explained that he had been planning to visit a member of his videogame community that lives in another country once the Covid-19 restrictions was lifted. The participant’s statement shows that conversations can go beyond videogame related topics. In addition, the quote indicates that, one can experience a unity through playing videogames, which can lead to friendship regardless of different geographical locations.

Some of the participants thought it sometimes was easier to be social on virtual platforms than in real life, as they were more comfortable in communicating online and were able to bond over a common interest in videogames. Nevertheless, most of the participants thought that overall, it was better to meet face-to-face than virtually, if that was an option. They described playing videogames virtually as a different experience than being physically together in the same room:

If we play the same videogame online or if we physically bring the PC and have a LAN (physical gathering for playing videogames)—then there will be a much better atmosphere on the LAN than if I was home alone (Participant 7).

The participants described that in the big picture, virtual socialization cannot replace meeting face-to-face. They explained that being in the same room leads to better communication and presence in the situation, and that there is more of the same focus when meeting physically:

When you play online, you focus on so many other things as well. I check my phone, maybe have a conversation on Messenger and check online newspapers at the same time. But, if you are gathered physically, you feel that you have the same focus (Participant 4).

Still, most participants agreed that if the alternative was not to be social at all, being virtually social through videogames was far better: “*It is absolutely better than nothing, sometimes you take what you can get”* (Participant 1).

## 4 Discussion

The findings of this study show that playing videogames can be a healthy social activity, which can be used as a method for both keeping in touch with friends and family and expanding social networks. In this study, the participants consistently reported that they observed an increase in the active usage of video games as a means of socializing with friends and family during the pandemic. They shared that a larger number of individuals they interacted with were also engaging in video game usage for social purposes. This finding is in line with Kim and Lee who highlights the growing prevalence of video game usage during the Covid-19 pandemic based on reports of individuals' experiences and perspectives (39). Marston and colleagues suggest that the benefits of virtual socialization, inherent in online videogames, could be used as a method to decrease some of the negative factors caused by the pandemic, such as stress, depression and a sense of loneliness (40). The use of virtual socialization to counteract loneliness is relevant also for non-pandemic times, and is perhaps particularly relevant for persons who face chronic barriers to socialization, such as those with physical disabilities (41).

The findings show that the participants were sometimes unable to meet with friends and family physically due to factors such as neuropathic pain, hospital admissions and the fear of being a burden to others in physical activities. The participants stated that because of these obstacles, they chose to socialize through online videogames. In accordance with the salutogenic approach, the choice to use videogames as an arena for socialization can be seen as the ability to use the resistance resources in themselves and in their surroundings. The participants were able to see videogames as a resource and were therefore able to partially or even fully fulfill their social needs. One of the participants was injured as a young child. He experienced a lack of social inclusion due to his use of a wheelchair. Even though videogames were not common technology in every household when he was young, he still recognized videogames as a resource that he could utilize to meet his social needs. An individual's ability to identify and use resources both in themselves and in their surroundings indicates a strong sense of coherence (42). Furthermore, a strong sense of coherence can contribute to the participant believing that their situation is manageable and may lead to good health instead of stress that can lead to a sense of loneliness and/or isolation. However, not everyone has the same resources, the same amount of knowledge regarding technology or is aware of the possibility of using videogames as an arena to socialize. In these situations, the responsibility to facilitate virtual socialization might fall onto others in their close vicinity, such as friends, family, teachers or healthcare professionals (2). Yet, the participants shared that their facilitators often lacked knowledge regarding the use of videogames as a social arena or had negative attitudes towards the method.

The participants acknowledged the need to be social and interact with friends and family, especially in a situation where their lives had changed due to their SCI. They stated that during their post-acute rehabilitation, there was no focus on facilitating the use of videogames and that the possibilities to such exposure within the hospital was limited. Including online video games as a part of rehabilitation could contribute to increase patientś social participation during hospital admissions, which has significant clinical implications. Facilitation of playing videogames from healthcare professionals could enable participants to interact with friends and family and possibly contribute to their online community. Furthermore, participants emphasized a need for social support after their injury. Social support can be attained by having a sense of belonging to a social network (43), which facilitation of playing online videogames could contribute towards. In addition, persons with SCI have highlighted the importance of regaining a sense of normality after the injury (44). Misztal reported that normality was experienced when taking part in everyday activities (45). As playing videogames is a popular everyday leisure activity worldwide (9), facilitation of this activity may lead to a sense of normality and increased social participation for many. According to the 2018–2019 Norwegian public health report, technical aids can be used to increase social interactions for people that for various reasons are not able to participate in social activities and take part in social life in the same manner as others (5). Rico-Uribe and colleagues emphasized that a lack of social support and loneliness increases the risk of disease and depression and weakens the ability to cope with stressors (46).

It is possible that the stress and insecurities that some may experience in hospital discharge and resuming contact with friends or acquaintances can be reduced through social contact via videogames during hospital admission. In this way, videogames can be a resource to help facilitate the transition from the hospital back to everyday life. Guilcher and colleagues accentuated that facilitating activities that can lead to social interaction and participation in society and help promote good health and well-being should be a subject of interest for healthcare professionals (2). However, healthcare professionals do not usually facilitate videogames within healthcare as they do with other leisure activities. To optimize facilitation and maximize benefits, healthcare providers should acquire knowledge regarding various videogames that are suited for social interaction and learn how to adapt videogame equipment to individual needs. This will require time and financial resources.

The participants expressed that videogames were not used only as entertainment, but as an important activity that helped meet their social needs. Maslow's hierarchy of needs emphasizes the importance of social interaction and that the feeling of being part of a group can be seen as a basic human need (31). The participants shared various positive examples of online videogames as a social arena. However, excessive use of videogames might have a negative impact on other aspects of life. Maslow's hierarchy of needs presents basic needs for health and wellbeing, such as rest, food, water, security and safety. If these needs are not satisfied, the human body cannot function optimally (47). If the amount of resources spent on videogames interfere with fulfilling basic needs, this might be a sign of videogame addiction (48). In 2018, gaming disorder was included in the 11th revision of the International Classification of Diseases (ICD-11) (48). The diagnostic criteria for this disorder includes obsessive and compulsive overuse of online videogames, where the pattern of gaming behavior results in distress or significant impairment in personal, family, social, educational, occupational or other important areas of functioning (48).

However, the establishment of the disorder has been criticized for having a poor evidence-base, and it has been argued that the inclusion of videogame addiction in ICD-11 is premature (49). Aarseth and colleagues emphasized that the majority of gamers will be affected negatively by this classification, including those who play videogames as a part of a normal and healthy life (49). However, to minimize the potential harms of playing videogames, such as addiction and lost face-to-face interaction, healthcare providers should be aware of possible signs that basic needs are not being fulfilled. In our sample, the participants did not report any unhealthy effects of playing videogames. However we did not screen this systematic, which might have been preferable.

Our participants expressed that physically meeting to play videogames was preferable to online meetings, describing it as a more socially focused setting than playing online. In Norway today, few physical arenas for playing videogames exist. The Norwegian Ministry of Culture and Equality has published a guide on computer culture and how to establish meeting places for playing videogames, where they state that physically meeting to play videogames can create an arena for reflection, communication and a sense of belonging (50). A public health report from 2018–19 states that new universally designed meeting places should be established to prevent loneliness and enable physical socialization (5). One possible physical meeting place that could contribute to meeting these goals could be a wheelchair accessible arena for playing videogames. This can also be seen in reference to the taxonomy of social participation proposed by Levasseur and colleagues (8), as participants would play videogames together, interact with each other while playing and also contribute to both a physical community of others with physical disabilities and the larger web-based community of fellow gamers (8).

The participants in this study had played videogames for a long time and seemed to be able to manage balancing their time spent playing videogames with other aspects of their lives, such as school and work. Some had played with friends and siblings while others had competed in e-sports. This demonstrated a great variation of experiences. Despite this variation, one common denominator was that all participants had experienced negative attitudes from others related to their use of videogames. Negative attitudes towards videogames can be attributed to multiple factors, such as media's negative portrayal of videogames and the users' misconceptions and lack of experience (19).

However, the participants also described experiencing a change in attitudes over the past few years. This is also reflected in society in multiple ways. Videogames have gained increased focus in schools, where they are used to teach basic skills such as reading, writing, mathematics and oral skills (51). Moreover, playing videogames has become one of the most common leisure activities in the world (9). The change in attitude is also prevalent in research, which has lately taken a more neutral stance in which both positive and negative factors of the activity are investigated (14–16, 21, 40, 52, 53). In addition, society has been greatly digitalized over the past decade, with more focus on technology in everyday activities.

### 4.1 Strengths and limitations

This study fills a knowledge gap as it qualitatively explores a topic that has gained little attention in rehabilitation research. However, the study also has some limitations. We only performed one focus group interview with seven participants. The Covid-19 pandemic period may have affected the recruitment of participants, which can be attributed to the decline in patient admissions at the Rehabilitation Hospital during this period. Seven participants is within recommendations for number of participants in a focus group. However, it would have been preferable to perform more than one focus group to ensure saturation and enhance the validity of the data material. In addition, it would have been preferable with a sample including woman, a broader age span and more diverse physical functioning to ensure diversity in the sample. This would have enabled further exploration of possible differences in experiences between or across patient groups. Due to the inclusion criteria of having prior experience of videogames as a mean for social participation, the possibility of participants being more positive towards the method than the general population was present. This may have led to a bias in participant reports. On the other hand, the research question was related to understanding experiences from those who have played videogames, which requires a sample with this experience.

When acknowledging these limitations, it is important to note that the results may have limited generalizability and the data collected might lack certain crucial aspects. Nevertheless, despite the small sample size, the data obtained was rich in content. This reinforces our belief in the importance of the study's theme and the necessity to shed light on the participants' experiences with using videogames as a mean of socialization. Furthermore, a notable strength of this current study is its specific focus on how participants perceive virtual social participation.

## 5 Conclusion

The findings of this study are in line with the few studies previously published, in showing that playing videogames has the potential to be an arena for social participation. The findings show that the participants have played online videogames as a way of maintaining and expanding their social network, and they especially emphasized that playing videogames was an important means of socialization during the pandemic. Furthermore, the findings show that while playing videogames may contribute to fulfilling social needs and therefore is a positive means towards socialization, it cannot fully replace physical interactions and engagement. Videogames are thus more than mere tools for entertainment.

One of the main findings of this study is that the participants were not largely exposed to the facilitation of playing videogames during their post-acute rehabilitation. If facilitation of videogames is encouraged in rehabilitation centers, individuals could partially or even fully fulfill their social needs during hospital admissions as well as in a home setting. The facilitation of using videogames as a method social participation may also ease the transition from the hospital to everyday life.

Further research should focus on how factors such as age, gender or level of physical functioning could affect how videogames as a method of social participation are experienced. In addition, exploration of whether the use of original or adaptive controllers may affect the accessibility or usability is needed.

Healthcare professionals should recognize that social interactions can take place on virtual platforms, especially with modern society's increased focus on technology. Healthcare professionals should strive to gain knowledge on how to facilitate any activity that can lead to social interaction and participation in society, thereby encouraging good health and well-being.

## Data Availability

The raw data supporting the conclusions of this article will be made available by the authors, upon reasonable request.
